# Supplementary material for: Characterization and Safety Evaluation of Autoclaved Gut Commensal Parabacteroides goldsteinii RV-01
Source: Int J Mol Sci. 2024 Nov 25;25(23):12660. doi: 10.3390/ijms252312660 (PMC11641655; doi:10.3390/ijms252312660)
Supplement: Supplementary file 1 [file ijms-25-12660-s001.zip › Supplementary Tables 20241124.pdf]

Supplementary Table S1. Revertant colonies of *Salmonella* strain TA97a in bacterial reverse mutation test

Without S9

Group (mg/plate)	Number of revertants/Plate			Mean ± S.D.
	No. 1	No. 2	No. 3	
5	90	100	89	93.0 ± 6.1
2.5	99	88	91	92.7 ± 5.7
1.25	88	108	84	93.3 ± 12.9
0.625	89	83	86	86.0 ± 3.0
0.3125	90	92	84	88.7 ± 4.2
Negative control [a]	86	90	95	90.3 ± 4.5
Positive control	390	381	345	372.0 ± 23.8 [#]

With S9

Group (mg/plate)	Number of revertants/Plate			Mean ± S.D.
	No. 1	No. 2	No. 3	
5	106	117	101	108.0 ± 8.2
2.5	100	106	105	103.7 ± 3.2
1.25	102	102	103	102.3 ± 0.6
0.625	104	107	100	103.7 ± 3.5
0.3125	100	104	107	103.7 ± 3.5
Negative control [a]	119	109	104	110.7 ± 7.6
Positive control	497	547	503	515.7 ± 27.3 [#]

Data represent the mean±standard deviation of triplicate determinations.

[#] The average number of revertant colonies was increased more than two times as compared to that of the negative control.

[a] Sterile water was used as a negative control.

Supplementary Table S2. Revertant colonies of *Salmonella* strain TA98 in bacterial reverse mutation test

Without S9

Group (mg/plate)	Number of revertants/Plate			Mean ± S.D.
	No. 1	No. 2	No. 3	
5	20	18	17	18.3 ± 1.5
2.5	19	18	11	16.0 ± 4.4
1.25	14	18	16	16.0 ± 2.0
0.625	15	19	16	16.7 ± 2.1
0.3125	19	14	16	16.3 ± 2.5
Negative control [a]	18	20	15	17.7 ± 2.5
Positive control	532	689	581	600.7 ± 80.3 [#]

With S9

Group (mg/plate)	Number of revertants/Plate			Mean ± S.D.
	No. 1	No. 2	No. 3	
5	25	24	21	23.3 ± 2.1
2.5	21	16	20	19.0 ± 2.6
1.25	26	28	24	26.0 ± 2.0
0.625	26	20	17	21.0 ± 4.6
0.3125	21	20	19	20.0 ± 1.0
Negative control [a]	26	20	21	22.3 ± 3.2
Positive control	115	129	189	144.3 ± 39.3 [#]

Data represent the mean±standard deviation of triplicate determinations.

[#] The average number of revertant colonies was increased more than two times as compared to that of the negative control.

[a] Sterile water was used as a negative control.

Supplementary Table S3. Revertant colonies of *Salmonella* strain TA100 in bacterial reverse mutation test

Without S9

Group (mg/plate)	Number of revertants/Plate			Mean ± S.D.
	No. 1	No. 2	No. 3	
5	86	79	85	83.3 ± 3.8
2.5	90	85	95	90.0 ± 5.0
1.25	83	89	93	88.3 ± 5.0
0.625	88	80	79	82.3 ± 4.9
0.3125	84	83	92	86.3 ± 4.9
Negative control [a]	83	86	94	87.7 ± 5.7
Positive control	329	382	420	377.0 ± 45.7 [#]

With S9

Group (mg/plate)	Number of revertants/Plate			Mean ± S.D.
	No. 1	No. 2	No. 3	
5	99	83	96	92.7 ± 8.5
2.5	93	80	88	87.0 ± 6.6
1.25	106	108	108	107.3 ± 1.2
0.625	95	88	87	90.0 ± 4.4
0.3125	91	92	103	95.3 ± 6.7
Negative control [a]	113	88	93	98.0 ± 13.2
Positive control	404	305	307	338.7 ± 56.6 [#]

Data represent the mean±standard deviation of triplicate determinations.

[#] The average number of revertant colonies was increased more than two times as compared to that of the negative control.

[a] Sterile water was used as a negative control.

Supplementary Table S4. Revertant colonies of *Salmonella* strain TA102 in bacterial reverse mutation test

Without S9

Group (mg/plate)	Number of revertants/Plate			Mean ± S.D.
	No. 1	No. 2	No. 3	
5	373	376	419	389.3 ± 25.7
2.5	383	381	384	382.7 ± 1.5
1.25	389	375	383	382.3 ± 7.0
0.625	383	407	400	396.7 ± 12.3
0.3125	393	389	374	385.3 ± 10.0
Negative control [a]	388	389	407	394.7 ± 10.7
Positive control	1499	1516	1630	1548.3 ± 71.2 [#]

With S9

Group (mg/plate)	Number of revertants/Plate			Mean ± S.D.
	No. 1	No. 2	No. 3	
5	392	446	450	429.3 ± 32.4
2.5	423	453	463	446.3 ± 20.8
1.25	395	413	409	405.7 ± 9.5
0.625	426	406	384	405.3 ± 21.0
0.3125	464	386	440	430.0 ± 39.9
Negative control [a]	406	420	435	420.3 ± 14.5
Positive control	996	1001	1024	1007.0 ± 14.9 [#]

Data represent the mean±standard deviation of triplicate determinations.

[#] The average number of revertant colonies was increased more than two times as compared to that of the negative control.

[a] Sterile water was used as a negative control.

Supplementary Table S5. Revertant colonies of *Salmonella* strain TA1535 in bacterial reverse mutation test

Without S9

Group (mg/plate)	Number of revertants/Plate			Mean ± S.D.
	No. 1	No. 2	No. 3	
5	7	10	9	8.7 ± 1.5
2.5	13	12	9	11.3 ± 2.1
1.25	12	15	13	13.3 ± 1.5
0.625	9	8	14	10.3 ± 3.2
0.3125	9	14	19	14.0 ± 5.0
Negative control [a]	15	10	7	10.7 ± 4.0
Positive control	216	221	201	212.7 ± 10.4 [#]

With S9

Group (mg/plate)	Number of revertants/Plate			Mean ± S.D.
	No. 1	No. 2	No. 3	
5	10	16	12	12.7 ± 3.1
2.5	13	13	12	12.7 ± 0.6
1.25	12	14	12	12.7 ± 1.2
0.625	10	7	13	10.0 ± 3.0
0.3125	11	12	10	11.0 ± 1.0
Negative control [a]	9	11	13	11.0 ± 2.0
Positive control	166	126	218	170.0 ± 46.1 [#]

Data represent the mean±standard deviation of triplicate determinations.

[#] The average number of revertant colonies was increased more than three times as compared to that of the negative control.

[a] Sterile water was used as a negative control.

Supplementary Table S6. The percentage of cells with abnormalities in chromosome structure in *in vitro* mammalian chromosomal aberration test

Groups (mg/mL)	Cells with chromosomal aberrations (%)					
	3-hour treatment without S9		18-hour treatment without S9		3-hour treatment with S9	
	Structural aberrations	Numerical aberrations	Structural aberrations	Numerical aberrations	Structural aberrations	Numerical aberrations
5	0.0	0.0	0.0	0.0	0.3	0.3
2.5	0.7	0.0	0.0	0.0	0.3	0.3
1.25	0.0	0.0	0.3	0.0	0.0	0.0
0.625	0.0	0.0	0.3	0.0	0.7	0.3
0.3125	0.0	0.0	0.0	0.0	0.3	0.0
Negative control[a]	0.3	0.3	0.3	0.0	0.3	0.3
Positive control[b,c]	14.0*	0.0	13.0*	0.0	12.0*	0.0

n/a: not available
[a] Culture medium
[b] Positive control: 2 μM mitomycin C
[c] Positive control: 80 μM cyclophosphamide monohydrate
* Positive response: significant increases compared with the negative control (p<0.05)

Supplementary Table S7. The number of micronucleated reticulocytes in *in vivo* micronucleus assay

Group	Time point of blood collection (hour)	Individual data (MN/4000 RET)					Mean ± S.D.[a]	‰[#]
		No. 1	No. 2	No. 3	No. 4	No. 5		
Negative control	46~48	4	4	4	4	4	4.0 ± 0.0	1.00
Positive control	46~48	28	33	39	39	40	35.8 ± 5.2*	8.95*
Low dose (500 mg/kg)	46~48	5	2	2	4	2	3.0 ± 1.4	0.75
Middle dose (1000 mg/kg)	46~48	5	3	1	1	3	2.6 ± 1.7	0.65
High dose (2000 mg/kg)	46~48	4	2	3	3	6	3.6 ± 1.5	0.90

[a] Mean ± S.D., n = 5 (n means numbers of sample)
MN: micronucleus; RET: reticulocyte
* Significant difference in comparison with the negative control group ($p < 0.05$)
[#](MN/4000 RET)×1000

Supplementary Table S8. The number of reticulocytes in *in vivo* micronucleus assay

Group	Time point of blood collection (hour)	Individual data (RET/2000 RBC)					Mean ± S.D. [a]
		No. 1	No. 2	No. 3	No. 4	No. 5	
Negative control	46~48	66	74	56	69	63	65.6 ± 6.7
Positive control	46~48	31	34	44	34	34	35.4 ± 5.0
Low dose (500 mg/kg)	46~48	71	57	56	62	61	61.4 ± 5.9
Middle dose (1000 mg/kg)	46~48	64	47	71	66	59	61.4 ± 9.1
High dose (2000 mg/kg)	46~48	57	66	59	69	66	63.4 ± 5.1

[a] Mean ± S.D., n = 5 (n means numbers of sample)
RET: reticulocyte; RBC: red blood cell

Supplementary Table S9. Body weights of each group in both the main and recovery studies of 28-day repeated dose of oral subacute toxicity study

Main study male group

Group†	Dose (mg/kg)	Body weight (g)				
		Day 0‡	Day 7	Day 14	Day 21	Day 28
C	0	224.8 ± 9.9	287.6 ± 19.5	333.0 ± 26.1	370.4 ± 27.8	393.4 ± 26.9
TA-L	74	218.2 ± 9.3	281.0 ± 11.9	336.4 ± 15.6	380.6 ± 15.7	400.8 ± 18.9
TA-M	370	216.8 ± 10.6	272.4 ± 22.0	322.8 ± 29.1	362.6 ± 32.9	382.2 ± 30.6
TA-H	1500	226.4 ± 13.8	290.2 ± 20.1	340.6 ± 29.3	381.8 ± 34.2	401.4 ± 41.9

Main study female group

Group†	Dose (mg/kg)	Body weight (g)				
		Day 0‡	Day 7	Day 14	Day 21	Day 28
C	0	176.4 ± 9.0	201.8 ± 11.8	222.0 ± 16.7	238.2 ± 15.4	241.6 ± 19.2
TA-L	74	176.4 ± 8.6	206.6 ± 9.7	228.4 ± 14.8	239.0 ± 14.4	244.4 ± 9.9
TA-M	370	177.8 ± 4.6	201.8 ± 9.8	216.4 ± 11.9	229.8 ± 8.7	232.2 ± 8.3
TA-H	1500	175.4 ± 7.9	203.6 ± 5.8	220.8 ± 7.4	234.0 ± 9.5	240.0 ± 7.4

Recovery study male group

Group†	Dose (mg/kg)	Body weight (g)						
		Day 0‡	Day 7	Day 14	Day 21	Day 28	Day 35	Day 42
Re-C	0	232.4 ± 9.3	299.8 ± 11.0	355.0 ± 9.8	397.4 ± 14.0	431.4 ± 16.9	463.8 ± 17.7	478.4 ± 19.4
Re-H	1500	219.0 ± 8.3*	282.6 ± 14.7	333.0 ± 20.7	371.6 ± 24.4	403.6 ± 26.7	434.2 ± 28.6	443.6 ± 27.8

Recovery study female group

Group†	Dose (mg/kg)	Body weight (g)						
		Day 0‡	Day 7	Day 14	Day 21	Day 28	Day 35	Day 42
Re-C	0	178.0 ± 3.9	205.4 ± 9.3	224.4 ± 8.7	235.6 ± 15.7	244.8 ± 15.4	258.8 ± 15.5	257.8 ± 15.6
Re-H	1500	174.4 ± 11.3	198.2 ± 17.9	218.0 ± 18.2	234.6 ± 18.4	248.2 ± 19.6	258.0 ± 22.7	261.8 ± 23.1

Data were presented as mean ± S.D. of 5 animals per group.

* Significant difference compared to the control group ($p < 0.05$).

† C: control; TA-L: low dose group; TA-M: middle dose group; TA-H: high dose group; Re-C: recovery control group; Re-H: recovery high dose group.

‡ Day 0: the day prior to administration of the test article or sterile water (control).

Supplementary Table S10. Body weight of each individual male rat in 28-day repeated dose of oral subacute toxicity study

Group†	Dose (mg/kg)	Animal I.D.	Body weight (g)						
			Week 0#	Week 1	Week 2	Week 3	Week 4	Week 5	Week 6
C	0	239571101	214	268	311	349	380	-	-
		239571102	222	277	316	344	364	-	-
		239571103	222	291	351	391	407	-	-
		239571104	225	283	317	360	383	-	-
		239571105	241	319	370	408	433	-	-
TA-L	74	239571106	206	274	322	360	372	-	-
		239571107	216	274	335	378	399	-	-
		239571108	218	278	330	379	399	-	-
		239571109	219	277	332	382	411	-	-
		239571110	232	302	363	404	423	-	-
TA-M	370	239571111	206	244	282	315	341	-	-
		239571112	211	258	318	366	385	-	-
		239571113	211	279	331	372	390	-	-
		239571114	225	280	320	354	370	-	-
		239571115	231	301	363	406	425	-	-
TA-H	1500	239571116	206	257	292	323	328	-	-
		239571117	222	297	365	410	433	-	-
		239571118	228	290	336	384	410	-	-
		239571119	233	296	351	398	419	-	-
		239571120	243	311	359	394	417	-	-
Re-C	0	239571121	220	290	350	398	434	463	474
		239571122	229	295	346	385	420	452	470
		239571123	244	316	368	418	459	492	512
		239571124	230	292	348	384	416	446	462
		239571125	239	306	363	402	428	466	474
Re-H	1500	239571126	211	264	303	335	364	389	399
		239571127	214	279	329	372	405	438	446
		239571128	216	277	330	366	399	431	444
		239571129	222	290	344	384	412	446	454
		239571130	232	303	359	401	438	467	475

# Week 0: the first day of the study prior to administration.
† C: control; TA-L: low dose group; TA-M: middle dose group; TA-H: high dose group; Re-C: recovery control group; Re-H: recovery high dose group

Supplementary Table S11. Body weight of each individual female rat in 28-day repeated dose of oral subacute toxicity study

Group[†]	Dose (mg/kg)	Animal I.D.	Body weight (g)						
			Week 0[#]	Week 1	Week 2	Week 3	Week 4	Week 5	Week 6
C	0	239571201	168	187	207	221	222	-	-
		239571202	169	196	212	226	227	-	-
		239571203	173	205	219	241	242	-	-
		239571204	185	219	250	260	271	-	-
		239571205	187	202	222	243	246	-	-
TA-L	74	239571206	168	195	213	222	234	-	-
		239571207	170	202	223	231	241	-	-
		239571208	174	210	227	245	247	-	-
		239571209	181	205	226	237	240	-	-
		239571210	189	221	253	260	260	-	-
TA-M	370	239571211	171	186	207	222	225	-	-
		239571212	177	210	232	244	245	-	-
		239571213	177	199	216	224	227	-	-
		239571214	183	206	203	228	228	-	-
		239571215	181	208	224	231	236	-	-
TA-H	1500	239571216	167	202	213	227	240	-	-
		239571217	170	205	215	224	237	-	-
		239571218	174	196	220	231	232	-	-
		239571219	179	203	225	243	252	-	-
		239571220	187	212	231	245	239	-	-
Re-C	0	239571221	175	197	220	224	242	252	250
		239571222	173	201	224	236	233	251	255
		239571223	179	199	213	218	232	248	243
		239571224	182	219	236	258	270	286	284
		239571225	181	211	229	242	247	257	257
Re-H	1500	239571226	159	176	193	216	228	228	229
		239571227	173	187	206	216	228	242	250
		239571228	175	201	225	238	251	262	264
		239571229	174	204	228	245	267	280	281
		239571230	191	223	238	258	267	278	285

[#] Week 0: the first day of the study prior to administration.

[†] C: control; TA-L: low dose group; TA-M: middle dose group; TA-H: high dose group; Re-C: recovery control group; Re-H: recovery high dose group

Supplementary Table S12. Organ weights of male rats in the main study of 28-day repeated dose of oral subacute toxicity study

Male rats

	Group†	C	TA-L	TA-M	TA-H
	Dose (mg/kg)	0	74	370	1500
Organ	Unit				
Brain	Weight (g)	2.004 ± 0.043	2.024 ± 0.019	2.002 ± 0.050	1.986 ± 0.119
Adrenals	Weight (g)	0.0622 ± 0.0094	0.0560 ± 0.0109	0.0554 ± 0.0028	0.0544 ± 0.0053
	Ratio (%)	3.1108 ± 0.5185	2.7668 ± 0.5348	2.7662 ± 0.0873	2.7504 ± 0.3474
Heart	Weight (g)	1.304 ± 0.147	1.256 ± 0.063	1.226 ± 0.058	1.240 ± 0.162
	Ratio	0.654 ± 0.086	0.620 ± 0.032	0.612 ± 0.042	0.624 ± 0.048
Kidneys	Weight (g)	3.222 ± 0.344	3.182 ± 0.217	3.050 ± 0.231	3.206 ± 0.481
	Ratio	1.612 ± 0.200	1.570 ± 0.109	1.528 ± 0.146	1.610 ± 0.193
Liver	Weight (g)	12.748 ± 1.674	12.608 ± 0.749	11.780 ± 1.333	12.678 ± 1.933
	Ratio	6.372 ± 0.932	6.228 ± 0.332	5.896 ± 0.777	6.354 ± 0.654
Spleen	Weight (g)	0.502 ± 0.178	0.568 ± 0.076	0.612 ± 0.049	0.580 ± 0.096
	Ratio	0.250 ± 0.091	0.282 ± 0.038	0.306 ± 0.029	0.292 ± 0.041
Thymus	Weight (g)	0.428 ± 0.056	0.514 ± 0.022	0.466 ± 0.094	0.478 ± 0.125
	Ratio	0.212 ± 0.024	0.254 ± 0.015	0.234 ± 0.050	0.240 ± 0.055
Epididymides	Weight (g)	0.994 ± 0.134	0.982 ± 0.092	0.910 ± 0.057	0.934 ± 0.174
	Ratio	0.496 ± 0.059	0.486 ± 0.049	0.456 ± 0.031	0.466 ± 0.068
Pro-Se-Co	Weight (g)	2.230 ± 0.268	2.106 ± 0.217	2.098 ± 0.298	2.214 ± 0.411
	Ratio	1.110 ± 0.128	1.044 ± 0.107	1.052 ± 0.168	1.108 ± 0.158
Testes	Weight (g)	3.124 ± 0.272	3.196 ± 0.118	3.202 ± 0.103	3.140 ± 0.304
	Ratio	1.560 ± 0.153	1.580 ± 0.068	1.604 ± 0.075	1.580 ± 0.098

Data were presented as mean ± S.D. of 5 animals per group.
Ratio: Organ weight/Brain weight; Ratio (%): (Organ weight/Brain weight) × 100%.
† C: control; TA-L: low dose group; TA-M: middle dose group; TA-H: high dose group.
Pro-Se-Co: Prostate-seminal vesicle with coagulating gland.

Supplementary Table S13. Organ weights of female rats in the main study of 28-day repeated dose of oral subacute toxicity study

Female rats

Organ	Group† / Dose (mg/kg) / Unit	C 0	TA-L 74	TA-M 370	TA-H 1500
Brain	Weight (g)	1.910 ± 0.104	1.906 ± 0.056	1.930 ± 0.091	2.000 ± 0.086
Adrenals	Weight (g)	0.0594 ± 0.0053	0.0694 ± 0.0034	0.0628 ± 0.0125	0.0594 ± 0.0071
	Ratio (%)	3.1128 ± 0.2640	3.6424 ± 0.1876	3.2736 ± 0.7552	2.9662 ± 0.2783
Heart	Weight (g)	0.850 ± 0.073	0.866 ± 0.077	0.818 ± 0.031	0.862 ± 0.058
	Ratio	0.444 ± 0.040	0.456 ± 0.043	0.428 ± 0.033	0.432 ± 0.039
Kidneys	Weight (g)	1.898 ± 0.195	1.890 ± 0.174	1.758 ± 0.113	1.830 ± 0.174
	Ratio	0.992 ± 0.068	0.992 ± 0.070	0.914 ± 0.090	0.916 ± 0.085
Liver	Weight (g)	7.628 ± 0.956	7.538 ± 0.742	7.074 ± 0.622	7.324 ± 0.457
	Ratio	3.986 ± 0.319	3.950 ± 0.281	3.682 ± 0.483	3.672 ± 0.315
Spleen	Weight (g)	0.424 ± 0.079	0.384 ± 0.046	0.360 ± 0.037	0.406 ± 0.072
	Ratio	0.220 ± 0.034	0.200 ± 0.019	0.188 ± 0.022	0.204 ± 0.036
Thymus	Weight (g)	0.378 ± 0.105	0.408 ± 0.069	0.352 ± 0.097	0.404 ± 0.034
	Ratio	0.198 ± 0.049	0.214 ± 0.032	0.186 ± 0.047	0.204 ± 0.017
Ovaries	Weight (g)	0.0774 ± 0.0132	0.0778 ± 0.0108	0.0702 ± 0.0108	0.0828 ± 0.0221
	Ratio (%)	4.0336 ± 0.4957	4.0788 ± 0.5144	3.6406 ± 0.5609	4.1300 ± 1.0090
Uterus	Weight (g)	0.472 ± 0.161	0.584 ± 0.300	0.526 ± 0.181	0.518 ± 0.192
	Ratio	0.246 ± 0.070	0.310 ± 0.166	0.274 ± 0.099	0.258 ± 0.091

Data were presented as mean ± S.D. of 5 animals per group.
Ratio: Organ weight/Brain weight; Ratio (%): (Organ weight/Brain weight) × 100%.
† C: control; TA-L: low dose group; TA-M: middle dose group; TA-H: high dose group.

Supplementary Table S14. Organ weights of male rats in the recovery study of 28-day repeated dose of oral subacute toxicity study

Male rats

	Group†	Re-C	Re-H
	Dose (mg/kg)	0	1500
Organ	Unit		
Brain	Weight (g)	2.082 ± 0.046	2.026 ± 0.054
Adrenals	Weight (g)	0.0650 ± 0.0112	0.0630 ± 0.0054
	Ratio (%)	3.1226 ± 0.5435	3.1146 ± 0.3191
Heart	Weight (g)	1.428 ± 0.058	1.394 ± 0.116
	Ratio	0.686 ± 0.036	0.688 ± 0.054
Kidneys	Weight (g)	3.796 ± 0.335	3.732 ± 0.513
	Ratio	1.820 ± 0.135	1.844 ± 0.275
Liver	Weight (g)	15.592 ± 1.101	14.054 ± 1.366
	Ratio	7.486 ± 0.485	6.938 ± 0.663
Spleen	Weight (g)	0.638 ± 0.046	0.652 ± 0.068
	Ratio	0.306 ± 0.027	0.322 ± 0.037
Thymus	Weight (g)	0.514 ± 0.095	0.400 ± 0.083
	Ratio	0.246 ± 0.044	0.200 ± 0.037
Epididymides	Weight (g)	1.278 ± 0.092	1.196 ± 0.094
	Ratio	0.614 ± 0.036	0.590 ± 0.058
Pro-Se-Co	Weight (g)	3.096 ± 0.617	2.738 ± 0.156
	Ratio	1.486 ± 0.302	1.352 ± 0.088
Testes	Weight (g)	3.410 ± 0.159	3.126 ± 0.244
	Ratio	1.640 ± 0.097	1.544 ± 0.142

Data were presented as mean ± S.D. of 5 animals per group.
Ratio: Organ weight/Brain weight; Ratio (%): (Organ weight/Brain weight) × 100%.
† Re-C: recovery control group; Re-H: recovery high dose group.
Pro-Se-Co: Prostate-seminal vesicle with coagulating gland.

Supplementary Table S15. Organ weights of female rats in the recovery study of 28-day repeated dose of oral subacute toxicity study

Female rats

	Group†	Re-C	Re-H
	Dose (mg/kg)	0	1500
Organ	Unit		
Brain	Weight (g)	1.960 ± 0.071	1.874 ± 0.061
Adrenals	Weight (g)	0.0702 ± 0.0078	0.0730 ± 0.0055
	Ratio (%)	3.5848 ± 0.4180	3.8978 ± 0.3028
Heart	Weight (g)	0.894 ± 0.098	0.866 ± 0.044
	Ratio	0.456 ± 0.042	0.464 ± 0.022
Kidneys	Weight (g)	2.012 ± 0.141	1.976 ± 0.137
	Ratio	1.028 ± 0.078	1.056 ± 0.099
Liver	Weight (g)	7.986 ± 0.992	7.840 ± 0.451
	Ratio	4.066 ± 0.378	4.184 ± 0.226
Spleen	Weight (g)	0.450 ± 0.065	0.432 ± 0.059
	Ratio	0.228 ± 0.031	0.230 ± 0.025
Thymus	Weight (g)	0.378 ± 0.069	0.344 ± 0.043
	Ratio	0.194 ± 0.043	0.184 ± 0.024
Ovaries	Weight (g)	0.0836 ± 0.0111	0.0906 ± 0.0138
	Ratio (%)	4.2760 ± 0.6488	4.8248 ± 0.6204
Uterus	Weight (g)	0.486 ± 0.127	0.694 ± 0.237
	Ratio	0.248 ± 0.064	0.368 ± 0.125

Data were presented as mean ± S.D. of 5 animals per group.
Ratio: Organ weight/Brain weight; Ratio (%): (Organ weight/Brain weight) × 100%.
† Re-C: recovery control group; Re-H: recovery high dose group.

Supplementary Table S16. Incidence of histopathological lesions in the main study of 28-day repeated dose of oral subacute toxicity study

Organ	Lesions#	Group†			
		Control		TA-H	
		♂	♀	♂	♀
Adrenal gland		-	-	-	-
Aorta		-	-	-	-
Brain		-	-	-	-
Fore		-	-	-	-
Middle		-	-	-	-
Cerebellum		-	-	-	-
Bone		-	-	-	-
Bone marrow		-	-	-	-
Cervix		N	-	N	-
Coagulative gland		-	N	-	N
Epididymis		-	N	-	N
Esophagus		-	-	-	-
Eyes		-	-	-	-
Harderian gland		-	-	-	-
Heart		-	-	-	-
Intestine, small		-	-	-	-
Duodenum		-	-	-	-
Jejunum		-	-	-	-
Ileum		-	-	-	-
Intestine, large		-	-	-	-
Caecum		-	-	-	-
Colon		-	-	-	-
Rectum		-	-	-	-
Kidney					
	Mineralization, tubule, multifocal, minimal to slight	-	2/5	-	4/5
Liver					
	Infiltration, mononuclear cell, focal, minimal to slight	-	1/5	-	1/5
Lung		-	-	-	-
Lymph node					
Cervical		-	-	-	-
Mesenteric		-	-	-	-
Mammary gland		-	-	-	-

Incidence: Affected rats/ Total examined rats (n = 5)

-: No significant lesions; N: No tissue available.

# Degree of lesions was graded from one to five depending on severity: 1 = minimal (< 1%); 2 = slight (1-25%); 3 = moderate (26-50%); 4 = moderate/severe (51-75%); 5 = severe/high (76-100%).

† C: control; TA-H: high dose group.

Organ	Lesions#	Group†			
		Control		TA-H	
		♂	♀	♂	♀
Optic nerve		-	-	-	-
Ovary		N	-	N	-
Oviduct		N	-	N	-
Pancreas		-	-	-	-
Parathyroid gland		-	-	-	-
Pituitary		-	-	-	-
Prostate gland			N		N
	Infiltration, mononuclear cell, focal, slight	2/5		1/5	
Salivary gland					
Mandibular lobe		-	-	-	-
Sublingual lobe		-	-	-	-
Sciatic nerve		-	-	-	-
Seminal vesicle		-	N	-	N
Skeletal muscle		-	-	-	-
Skin		-	-	-	-
Spinal cord					
Cervical		-	-	-	-
Lumbar		-	-	-	-
Thoracic		-	-	-	-
Spleen		-	-	-	-
Stomach		-	-	-	-
Testes		-	N	-	N
Thymus		-	-	-	-
Thyroid gland		-	-	-	-
Tongue		-	-	-	-
Trachea		-	-	-	-
Urinary bladder		-	-	-	-
Uterus		N	-	N	-
Vagina		N	-	N	-

Incidence: Affected rats/ Total examined rats (n = 5)

-: No significant lesions; N: No tissue available.

# Degree of lesions was graded from one to five depending on severity: 1 = minimal (< 1%); 2 = slight (1-25%); 3 = moderate (26-50%); 4 = moderate/severe (51-75%); 5 = severe/high (76-100%).

†C: control group; TA-H: high dose group.

Supplementary Table S17. Incidence of histopathological lesions of the main study (high dose groups) of 90-day repeated dose of oral subchronic toxicity study

Organ	Lesions†	Group			
		Control		TA-H #	
		♂	♀	♂	♀
Adrenal gland		-	-	-	-
Aorta		-	-	-	-
Brain					
Fore		-	-	-	-
Middle		-	-	-	-
Cerebellum		-	-	-	-
Bone		-	-	-	-
Bone marrow		-	-	-	-
Cervix		N	-	N	-
Coagulating gland		-	N	-	N
Epididymis		-	N	-	N
Esophagus		-	-	-	-
Eyes		-	-	-	-
Harderian gland					
	Infiltration, mononuclear cell, multifocal, slight to moderate	2/10	-	1/10	-
Heart		-	-	-	-
Intestine, small					
Duodenum		-	-	-	-
Jejunum		-	-	-	-
Ileum		-	-	-	-
Intestine, large					
Cecum		-	-	-	-
Colon		-	-	-	-
Rectum		-	-	-	-
Kidney					
	Cast, tubule, multifocal, minimal to slight	3/10	1/10	2/10	1/10
	Mineralization, tubule, multifocal, slight to moderate	1/10	6/10	-	4/10
Liver		-	-	-	-
Lung		-	-	-	-
Lymph node					
Cervical		-	-	-	-
Mesenteric		-	-	-	-
Mammary gland		-	-	-	-
Optic nerve		-	-	-	-
Ovary		N	-	N	-
Oviduct		N	-	N	-

Incidence: Affected rats/ Total examined rats (n = 10)
-: No significant lesions; N: No tissue available.
† Degree of lesions was graded from one to five depending on severity: 1 = minimal (< 1%); 2 = slight (1-25%); 3 = moderate (26-50%); 4 = moderate/severe (51-75%); 5 = severe/high (76-100%).
# TA-H: test article high dose group.

Organ	Lesions†	Group Control ♂	Control ♀	TA-H # ♂	TA-H # ♀
Pancreas		-	-	-	-
Parathyroid gland		-	-	-	-
Pituitary					
	Pseudocyst, pars intermedia, focal, moderate	1/10	-	-	-
Prostate gland					
	Infiltration, mononuclear cell, multifocal, slight	2/10	N	2/10	N
Salivary gland					
Mandibular lobe		-	-	-	-
Sublingual lobe		-	-	-	-
Sciatic nerve		-	-	-	-
Seminal vesicle		-	N	-	N
Skeletal muscle		-	-	-	-
Skin		-	-	-	-
Spinal cord					
Cervical		-	-	-	-
Lumbar		-	-	-	-
Thoracic		-	-	-	-
Spleen		-	-	-	-
Stomach		-	-	-	-
Testes		-	N	-	N
Thymus		-	-	-	-
Thyroid gland		-	-	-	-
Trachea		-	-	-	-
Urinary bladder		-	-	-	-
Uterus		N	-	N	-
Vagina		N	-	N	-

Incidence: Affected rats/ Total examined rats (n = 10)

-: No significant lesions; N: No tissue available.

† Degree of lesions was graded from one to five depending on severity: 1 = minimal (< 1%); 2 = slight (1-25%); 3 = moderate (26-50%); 4 = moderate/severe (51-75%); 5 = severe/high (76-100%).

# TA-H: test article high dose group.

Supplementary Table S18. Incidence of histopathological lesions of the recovery study (high dose group) of 90-day repeated dose of oral subchronic toxicity study

Organ		Lesions†	Group #			
			Re-C		Re-H	
			♂	♀	♂	♀
Adrenal gland			-	-	-	-
Aorta			-	-	-	-
Brain						
	Fore		-	-	-	-
	Middle		-	-	-	-
	Cerebellum		-	-	-	-
Bone			-	-	-	-
Bone marrow			-	-	-	-
Cervix			N	-	N	-
Coagulating gland			-	N	-	N
Epididymis			-	N	-	N
Esophagus			-	-	-	-
Eyes			-	-	-	-
Harderian gland			-	-	-	-
Heart			-	-	-	-
Intestine, small						
	Duodenum		-	-	-	-
	Jejunum		-	-	-	-
	Ileum		-	-	-	-
Intestine, large						
	Cecum		-	-	-	-
	Colon		-	-	-	-
	Rectum		-	-	-	-
Kidney						
		Cast, tubule, multifocal, minimal to slight	1/5	-	1/5	-
		Mineralization, tubule, multifocal, slight	1/5	2/5	-	2/5
Liver			-	-	-	-
Lung			-	-	-	-
Lymph node						
	Cervical		-	-	-	-
	Mesenteric		-	-	-	-
Mammary gland			-	-	-	-
Optic nerve			-	-	-	-
Ovary			N	-	N	-
Oviduct			N	-	N	-
Pancreas			-	-	-	-
Parathyroid gland			-	-	-	-
Pituitary						
		Cyst, pars distalis, focal, slight	-	1/5	-	-
Prostate gland			-	N	-	N

Incidence: Affected rats/ Total examined rats (n = 5)

-: No significant lesions; N: No tissue available.

† Degree of lesions was graded from one to five depending on severity: 1 = minimal (< 1%); 2 = slight (1-25%); 3 = moderate (26-50%); 4 = moderate/severe (51-75%); 5 = severe/high (76-100%).

# Re-C: recovery control group; Re-H: recovery high dose group.

Organ	Lesions†	Group #			
		Re-C		Re-H	
		♂	♀	♂	♀
Salivary gland					
Mandibular lobe		-	-	-	-
Sublingual lobe		-	-	-	-
Sciatic nerve		-	-	-	-
Seminal vesicle		-	N	-	N
Skeletal muscle		-	-	-	-
Skin		-	-	-	-
Spinal cord					
Cervical		-	-	-	-
Lumbar		-	-	-	-
Thoracic		-	-	-	-
Spleen		-	-	-	-
Stomach		-	-	-	-
Testes		-	N	-	N
Thymus		-	-	-	-
Thyroid gland		-	-	-	-
Trachea		-	-	-	-
Urinary bladder		-	-	-	-
Uterus		N	-	N	-
Vagina		N	-	N	-

Incidence: Affected rats/ Total examined rats (n = 5)

-: No significant lesions; N: No tissue available.

† Degree of lesions was graded from one to five depending on severity: 1 = minimal (< 1%); 2 = slight (1-25%); 3 = moderate (26-50%); 4 = moderate/severe (51-75%); 5 = severe/high (76-100%).

# Re-C: recovery control group; Re-H: recovery high dose group.
