# Supplementary material for: Characterization and Safety Evaluation of Autoclaved Gut Commensal Parabacteroides goldsteinii RV-01
Source: Int J Mol Sci. 2024 Nov 25;25(23):12660. doi: 10.3390/ijms252312660 (PMC11641655; doi:10.3390/ijms252312660)
Supplement: Supplementary file 1 [file ijms-25-12660-s001.zip › Supplementary Figures 20241122.pdf]

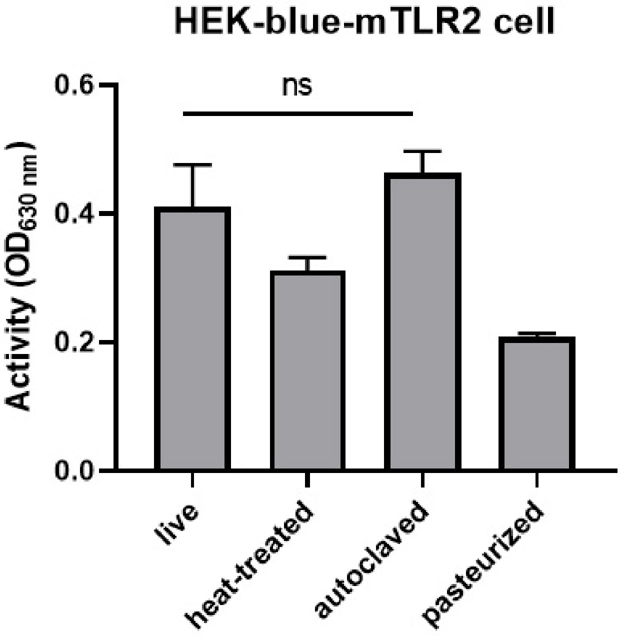

Supplementary Figure S1. Activities of *P. goldsteinii* RV-01 inactivated at different temperatures. The RV-01 was inactivated by heat-treated (100 °C for 15 min), autoclaved (121 °C for 15 minutes) or pasteurized (70 °C for 30 minutes). Activities of *P. goldsteinii* RV-01 inactivated at different temperatures co-cultured with HEK-blue-mTLR2 cells were compared.

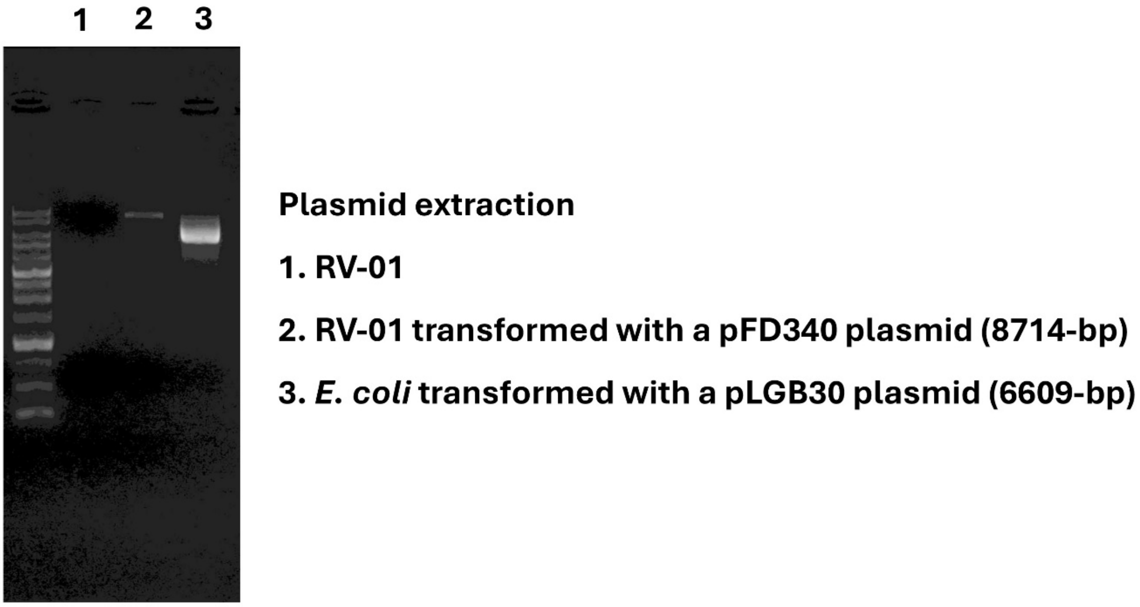

Supplementary Figure S2. *P. goldsteinii* RV-01 did not contain plasmid. To confirm there was no plasmid DNA present in *P. goldsteinii* RV-01, *P. goldsteinii* RV-01 and two controls harboring plasmids (*P. goldsteinii* RV-01 transformed with an 8714-bp pFD340 plasmid and *E. coli* transformed with a 6609-bp pLGB30 plasmid) were subjected for plasmid extraction. The result of agarose gel electrophoresis revealed that no plasmid was isolated from *P. goldsteinii* RV-01 whereas plasmid DNA with the expected size was detected in *P. goldsteinii* RV-01 transformed with a pFD340 plasmid and *E. coli* transformed with a pLGB30 plasmid.

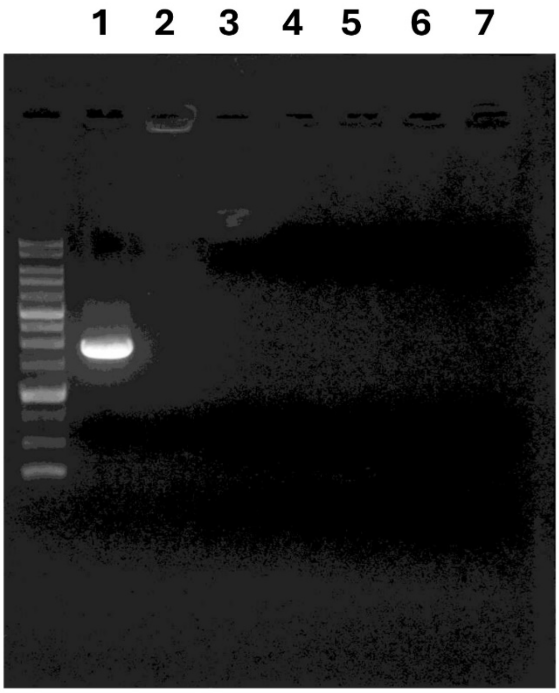

***tetQ* PCR**

1. RV-01 bacterial DNA

2. RV-01 ingredient (10^{10} TFU)

3. RV-01 ingredient (10^{9} TFU)

4. RV-01 ingredient (10^{8} TFU)

5. RV-01 ingredient (10^{7} TFU)

6. RV-01 ingredient (10^{6} TFU)

7. deionized water

Supplementary Figure S3. The *tetQ* DNA was not detected in the *P. goldsteinii* RV-01 ingredients. DNA was extracted from *P. goldsteinii* RV-01 ingredient, followed by PCR and agarose gel electrophoresis analysis.

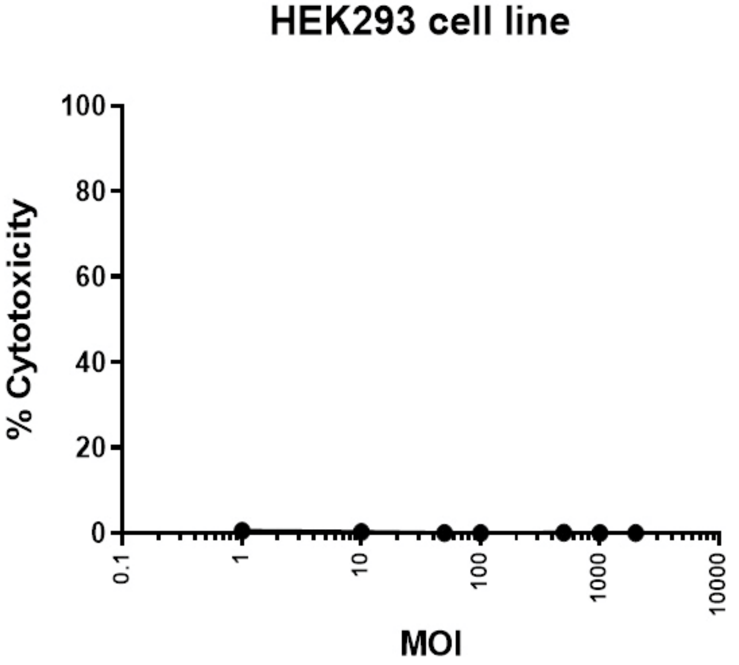

Supplementary Figure S4. ***P. goldsteinii*** **RV-01 did not show cytotoxicity activity against HEK 293 cells.** An *in vitro* cytotoxicity test of *P. goldsteinii* RV-01 on Human Embryonic Kidney Cells 293 (HEK293) was performed by using different multiplicity of infection (MOI) 1, 10, 50, 100, 500, 1000 and 2000. The black circle indicated the percentage of cytotoxicity against HEK 293 cells.
